# 

**Published:** 1886-11-20

**Authors:** 


### The Hospital.

An Institution, Family, and Parochial Journal of Hospitals, Asylums, and all Agencies for the Care of the Sick, Criticism and News.

SATURDAY, NOVEMBER 20TH, 1886.

### The Quilter Prizes.

Mr. W. Cuthbert Quilter, M.P., has offered £50 in prizes, with the view of encouraging all engaged in hospital and asylum work to promote efficiency and economy in the management of these institutions. The object of these Prizes, and a full description, will be found set forth on page 115 of The Hospital of the 13th inst., with the sections and subjects suggested. The following are the conditions and the subject of the first competition :—

The Conditions.

1. We shall offer two Prizes of varying amounts, according to the importance attached to the subject of the competition. A cheque will be sent on the day the paper is published in which the results of each competition are given.

2. In all cases the names and addresses of the competitors must be forwarded, even when a *nom de plume* is used.

3. The answers must be addressed to "The Prizes Editor, The Hospital, Norfolk House, Norfolk Street Strand," not later than the 15th December next, at noon. All communications must be written on one side of the paper only.

1. A Preliminary Competition.

At the outset we ask for suggestions from each of the before-mentioned classes of readers, as to the subjects in each section which they themselves consider to be the best to select for the purposes of this competition. The proposal which seems to us the most practical and in all respects the best will receive the first prize of £3, and the next best suggestion the second prize of £2.

### NOTICE TO CORRESPONDENTS.

All correspondence must be written on one side of the paper only.
We cannot undertake to return MSS. not used.

**Communications,** whether intended for insertion or for private information, must be authenticated by the names and addresses of the writers, not necessarily for publication.

**Local papers** containing reports or news paragraphs **should be marked.**

It is especially requested that early intelligence of **local events of interest,** or which it may be thought desirable to bring under notice, may be sent direct to the Editor.

Communications respecting editorial matters should be addressed to the Editor, Norfolk House Norfolk Street, Strand, London, W.C.

### The Children's Corner.

### OUR PASTIMES.

**Children's Quarterly Prize Competition.**

| | |
|---|---|
| Began .. .. .. .. .. | 2nd Oct., 1886 |
| Ends .. .. .. .. .. | 25th Dec., 1886 |

PRIZES.

| | |
|---|---|
| 1st .. .. .. .. .. | Thirty Shillings |
| 2nd .. .. .. .. .. | Twenty „ |
| 3rd .. .. .. .. .. | Ten „ |
| 4th .. .. .. .. .. | Five „ |

will be awarded to the four competitors who shall have sent in, during the thirteen weeks, the largest number of correct answers to our pastimes.

**Eighth Competition.**

No. 38. Draw the following figure without passing over any line twice and without lifting the pen.

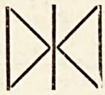

(Answers must show, by means of an arrow, the direction followed by the pen.)

No. 39. What creature in all probability entered the ark slowest, and what one quickest ?

No. 40. A palindrome is a word or sentence which reads the same either forwards or backwards as "redder," or the observation which Adam made to Eve when he first met her, "Madam, I'm Adam." Who can make a palindrome ?

No. 41. Translate

"Mea, mater, sus malum est."

No. 42. There was a certain king whose gardener had given him mortal offence. His majesty summoned him into his presence, and told him he must die, unless he successfully accomplished a task which the king thought was impossible. The gardener begged his majesty to state the task, and the king, answering him, replied : "Plant twelve trees so that they shall form six rows of four each." The gardener fulfilled the conditions, and so saved his life. How did he do it ? (The answer may be illustrated by 12 dots, representing the position of the trees.)

No. 43. Why is a tailor never to be found at home ?

**Results of Sixth Competition.**

No. 27. My little readers have not generally found this out. "What nation resembles Mr. Bass ?" To get the answer, ask yourself what Mr. Bass does. Why of course *He-brews* (He brews). Some of the guesses are very quaint. Master Jimmy, for instance, says that Mr. Bass being a "malter" (*sic*), he would suggest Malta as the answer. Possibly Master Jimmy was thinking of "maltster." Alsie thinks the Swiss must be the nation, because they have a town called "Basle" (Bass-all or all Bass), or perhaps the West Indians, because the chief town of St. Christopher is Basse-terre. I am glad to see that Alsie is such a clever little geographer.

No. 28. "Equus est in stabulo, et non est" is a little piece of genuine Latin. The catch lies in the second "est," which, in this case, is not "is," but the third person singular of the verb *edo*, to eat. Therefore, instead of a paradoxical assertion, it reads, "The horse is in the stable and does not *eat*." T. K. and Alsie alone have accurately guessed it.

No. 29. Why is "s" the most terrible letter in the alphabet ? Here my little friends have made some excellent guesses. "S," you know, is the beginning of *Sin*, *Strife*, and *Sorrow*, while, moreover, it invariably turns *laughter* into *slaughter*.

No. 30. *Boot* was easily converted into *Shoe*, thus : Boot, *soot*, *shot*, shoe.

No. 31. The key to the inscription is "E," which renders the sentence :

"Remember me, ye perfect men,
Ever keep these precepts ten."

No. 32. There are two or three ways in which the milkman might have measured off four pints. The eight-pint can being empty, and the five and three-pint cans full, the following figures will show how he might have acted :—

| | | | | | | | | | | | | | | | |
|---|---|---|---|---|---|---|---|---|---|---|---|---|---|---|---|
| 8 | 3 | 3 | 6 | 6 | 1 | 1 | or | 8 | 5 | 5 | 2 | 2 | 7 | 7 | Four |
| 0 | 5 | 2 | 2 | 0 | 5 | Four | | 0 | 0 | 3 | 3 | 5 | 0 | 1 | 1 |
| 0 | 0 | 3 | 0 | 2 | 2 | 3 | | 0 | 3 | 0 | 3 | 1 | 1 | 0 | 3 |

Ellie's solution was perhaps the best.

All right—Mikado.

Five right—Tina, Alsie, Ellie.

Four right—Florrie, Retsibsi, T. K., C. Tynget Jimmy.

Three right—Little Mary, Bismarck, Hetty Diosota, Ubique.

Answers, having the actual name and address (to which a pseudonym may be added for publication), must have "The Children's" Coupon attached (see advertisement pages), addressed to the "PUZZLE EDITOR," THE HOSPITAL, Norfolk House, Norfolk Street, Strand W.C., not later than Thursday, 25th November. Results will appear in our issue of the 4th December.

### "The Hospital" Charity Box.

OUR "Charity Box" is intended to afford a little practical help to the hospitals. Each week we propose a word for "anatomical dissection," and the first 150 competitors who extract the largest number of words from it receive the following prizes :—

| | |
|---|---|
| 1st .. .. .. | One Guinea. |
| 2nd .. .. .. | Half a Guinea. |
| 3rd .. .. .. | Five Shillings. |

The above Prizes will be repeated for every additional 150 competitors who may enter each week.

EIGHTH COMPETITION.

Compose the largest number of words you can from "SURGEON."

*Observe :* Proper names, abbreviations, foreign and obsolete words, and repetitions are barred.

Write only on one side of the paper, and total your words.

Append your real name and address, adding whether Mr., Mrs., or Miss, etc.

All replies, addressed "Charity Box," THE HOSPITAL, Norfolk House, Norfolk Street, W.C., must arrive by Friday, November 26th. Results will appear Saturday, Dec. 4th.

*Every Competitor is required to cut out and enclose with his list the "Charity Box" Coupon* (see advertisement pages), *and a shilling entrance fee* (postal orders only) *crossed* Lloyds, etc., Banking Company, Limited, THE HOSPITAL Account.

The proceeds resulting from each "Charity Box" competition will be funded until the end of December 1886. The total sum available for distribution, and the names of the hospitals selected for donations, will appear on Saturday, January 8, 1887. The distribution of the proceeds will be at the Editor's sole discretion.

RESULTS OF SIXTH COMPETITION

The "Charity Box" Competition has resulted as follows :—

| | Score. |
|---|---|
| Miss Marler, Berry Wood, nr. Northampton.. | 237—1st Prize. |
| Miss Louie Pepper, 37, Bethel Street, Norwich | 230—2nd Prize. |
| Miss J. Corbishley, The Byrons, Sutton, Macclesfield .. .. .. .. .. .. | 222—3rd Prize. |

The prizes have been forwarded to the winners.

As some of our readers are doubtless desirous of knowing the state of our "Charity Box" exchequer, we append the following result of the first six weeks' competitions :

| Weeks. | Competitors. | Entrance Fees. | Prizes awarded. |
|---|---|---|---|
| 1st | 21 | 1 1 0 | 1 16 6 |
| 2nd | 25 | 1 5 0 | 1 16 6 |
| 3rd | 30 | 1 10 0 | 1 16 6 |
| 4th | 30 | 1 10 0 | 1 16 6 |
| 5th | 49 | 2 9 0 | 1 16 6 |
| 6th | 36 | 1 16 0 | 1 16 6 |
| Literary Prize handed to "Charity Box".......... | | 0 10 6 | |
| | | £10 1 6 | £10 19 0 |

The balance thus far unhappily falls on the wrong side. We trust that our readers will second our efforts in this matter, taking part more generally in these competitions—which are certainly not devoid of interest and instruction—and by making our "Charity Box" known among their friends. Considering the great and rapidly increasing demand which there is for THE HOSPITAL, it was hoped competitors would have responded rather by hundreds than by tens.

**Notices to Competitors.**

"Kat."—You have mistaken the meaning of our rule, "repetitions barred." By this we mean, not only that similarly spelled words (as "act," verb, "act," a play, etc.) count only once, but also that the repetition of letters (unless they happen to repeat in the word set) is inadmissible. "Traitor," *e.g.*, cannot be made from "Charity Box," since it has two "t's" and two "r's."

Mr. C. I. Kirton.—(1), Scottish words are barred ; (2), actual obsoletes are not admitted whether they are found in classic authors or not ; (3), genuine compound words count ; (4), Anglicised foreign words such as "bric-à-brac," are permitted ; (5), American spellings are not allowed to duplicate a word ; for example, if "labor" only were obtainable from the word, it would count, but if both could be made, only one would count ; (6), see answer to Kat.

### BUSINESS COMMUNICATIONS AND ADVERTISEMENTS.

All communications concerning business matters, non-delivery of THE HOSPITAL, etc., should be addressed to the Manager, at the Publishing Offices, 30 and 32, Sardinia Street, Lincoln's Inn Fields, London, W.C.

**Cheap Advertisements** of the "Wanted" class, including those for situations, of Twenty-four Words or under, are charged 1*s.* an insertion, or three insertions for 2*s.* 6*d.*, but must be paid for in advance. Each additional line of Eight Words is 4*d.*

**Employers requiring assistants** are charged for one insertion of Twenty-four Words or under, 2*s.* 6*d.*, or 6*s.* for three insertions. Each additional line of Eight Words is 1*s.*

**Advertisements** for insertion in the weekly issue of THE HOSPITAL **must be delivered** at the Publishing Offices **not later than 11 a.m. on Wednesdays.**

### The In- and Out-patients' Hospital Diary.

*(Continued from p. 120.)*

This Diary is so arranged as to make some portion of it appear every week, and the whole in each monthly part. It has been very difficult to compile, and corrections will be very gratefully received at all times. It has been suggested that similar information about the larger Provincial and Scotch Hospitals would be useful to many readers. We should be glad to hear if this is felt to be the case.

### EYE DISEASES.—*Continued.*

| Hospitals and Terms of Admission. | Physicians, Surgeons, and Medical Officers, with Days and Hours of Attendance. |
|---|---|
| Royal South London Ophthalmic Hospital, 6, St. George's Circus, Southwark, S.E. Free to the necessitous poor; or by payment | *Surgeons*, Messrs. Watson, McHardy. Daily, 2 |
| Royal Westminster Ophthalmic Hospital, King William Street, W.C. Free to accidents and the indigent poor | *In- and Out-Surgeons*, Messrs. Power, M. F.; Frost, M. Th.; Rouse, Tu. S.; Hartridge, Tu. F.; Macnamara, M. Th.; Cowell, W. S.; Juler, W. S. Hour, 1 |
| St. Bartholomew's Hospital, Smithfield, E.C. See p. 16 | *Surgeons*, Messrs. Power, Th. 2.30; Vernon, Tu. S. 2.30 |
| St. George's Hospital. See p. 16 | *Surgeons*, Messrs. B. Carter, Frost, M. Th. 2; F. 1.15; W. S. 2 |
| St. Mary's Hospital. By Governors' letters. See p. 16 | *Surgeons*, Messrs. A. Critchett, H. Juler, Tu. F. 9.30 |
| St. Thomas's Hospital, Westminster Bdg. Rd., S.E. See p. 16 | *Surgeons*, Messrs. Nettleship, M. Th. F. 1.30; Lawford, M. W. 1.30 |
| University College Hospital. See p. 16 | *Surgeon*, Mr. Tweedy, M. Tu. Th. F. 2 |
| West London Hospital. See p. 16 | *Surgeons*, Messrs. B. Vernon, H. Dunn, Tu. Th. S. 2 |
| Westminster Hospital. See p. 16 | *Surgeon*, Mr. Cowell, M. Th. 2 |
| Western Ophthalmic Hospital, 153, Marylebone Road, W. By small payments | *In- and Out-Physicians*, Drs. Miller, M. 2; Collins, F. 2; Wheatly, M. Th. 2<br>*In-and Out-Surgeons*, Messrs. C. Jones, W. 2; Buxton, Tu. F. 2; Barrett, W. S. 2 |

### HEART, PARALYSIS, EPILEPSY, AND DISEASES OF THE NERVOUS SYSTEM GENERALLY.

| | |
|---|---|
| Hospital for Epilepsy and Paralysis, etc., 32, Portland Terrace, Regent's Park Road, N.W. By payment, but free to the necessitous poor | *In-and Out-Physicians*, Drs. J. Althaus, A. Bennett, G. Ogilvie, G. Laycock<br>*In- and Out-Surgeons*, Messrs. J. Bloxam, A. P. Gould, M. Tu. W. Th. F. 2 |
| National Hospital for the Paralysed and Epileptic, Queen Square, W.C. By subscriber's letter | *In- and Out-Physicians*, Drs. Ramskill, Radcliffe, H. Jackson, Buzzard, Ormerod, Beevor, M. Tu. W. F. 2.30<br>*In- and Out-Surgeons*, Messrs. W. Adams, Horsley, M. Tu. W. F. 1.30 |
| West End Hospital for Diseases of the Nervous System, 73, Welbeck St., W. By subscriber's letter | *In- and Out-Physicians*, Drs. Tibbitts, Stretch-Dowse, S. Armitage, M. Tu. W. Th. 1.30; F. 6 |

### ORTHOPÆDIC CASES AND DEFORMITIES OF BODY GENERALLY.

| | |
|---|---|
| City Orthopædic Hospital, 27, Hatton Garden, E.C. Free | *Surgeons*, Messrs. E. J. Chance, J. Addison, Tu. F. 2 |
| National Orthopædic Hospital, 234, Great Portland Street, W. Free | *In-Physician*, Dr. C. Beevor<br>*In-Surgeons*, Messrs. F. R. Fisher, W. Roeckel<br>*Out-Surgeon*, Mr. E. M. Little, M. Tu. Th. F. 2; W. 1 |
| Royal Orthopædic Hospital, 297, Oxford Street, W. By Governor's letter | *Surgeons*, Messrs. Broadhurst, H. Reeves, C. Read, W. Balkwill, H. F. Baker. Daily, 1 |
| St. Bartholomew's Hospital, Smithfield, E.C. See p. 16 | *Surgeon*, Mr. Walsham, M. 2.30 |
| St. George's Hospital, Hyde Park, S.W. See p. 16 | W. 1.30 |

### DISEASES OF THE SKIN.

| | |
|---|---|
| British Hospital for Diseases of the Skin (West Branch), 61, Great Marlborough St., W. By payment; necessitous free | *Surgeons*, Messrs. B. Squire, G. Gaskoin, M. 2, W. 7, F. 2 |
| Charing Cross Hospital. By Governor's letter. See p. 16 | *Physician*, Dr. Sangster, M. 2 |

### DISEASES OF THE SKIN.—*continued.*

| Hospitals and Terms of Admission. | Physicians, Surgeons, and Medical Officers, with Days and Hours of Attendance. |
|---|---|
| Guy's Hospital. By payment of 3*d.* each | *Physicians*, Drs. Pye-Smith, Hale White, Tu. 12 |
| Hospital for Diseases of the Skin, 52, Stamford St., S.E. Free and by payment | *Physician*, Dr. E. J. Payne<br>*Surgeons*, Messrs. Waren Tay and W. Cottle. (Out-patients seen M. W. 3, Tu. Th. F. 2) |
| King's College Hospital. See p. 16 | *Physician*, Dr. Duffin, F. 1 |
| London Hospital. See p. 16 | *Physician*, Dr. S. Mackenzie, Th. 9 |
| Middlesex Hospital. See p. 16 | *Physician*, Dr. R. Liveing, F. 4 |
| National Institution for Diseases of the Skin, 227, Gray's Inn Road, W.C. By payment, or free on certificate of a medical man or minister | *Physician*, Dr. B. Meadows, M. Th. 6 |
| North-West London Hospital. See p. 16 | *Physician*, Dr. J. Stowers, Tu. 1.30 |
| Paddington Green Children's Hospital. Free. See p. 32 | *Physician*, Dr. T. C. Fox, S. 9.15 |
| St. Bartholomew's Hospital. Free. See p. 16 | *Surgeon*, Mr. F. Cripps, F. 1.30 |
| St. George's Hospital. See p. 6 | *Physician*, Dr. Cavafy, W. 1.30 |
| St. Mary's Hospital. See p. 16 | *Surgeon*, Mr. Morris, M. Th. 9.30 |
| St. Thomas's Hospital. See p. 16 | *Physician*, Dr. Payne, W. 1.30 |
| University College Hospital. See p. 16 | *Physician*, Dr. Crocker, W. 1.30, S. 9.15 |
| Westminster Hospital. See p. 16 | *Physician*, Dr. T. C. Fox, W. 1.30 |

### STONE AND OTHER DISEASES OF URINARY ORGANS.

| | |
|---|---|
| London Hospital. By Governor's letter | Daily, 1 30. See *Surgeons*, p. 16 |
| St. Peter's Hospital for Stone, Henrietta Street, W.C. Admission free. Women and children only on Fridays | *Surgeons*, Messrs. E. H Fenwick, M. 2, S. 4 to 7; F. S. Edwards, M. 5 to 7, Th. 2; Heycock, Tu. 2, W. 5 to 7, F. 2 |

### DISEASES & IRREGULARITIES OF THE TEETH.

| | |
|---|---|
| Charing Cross Hospital. See p. 16 | *Surgeon*, Mr. Fairbank, M. W. F. 9.0 |
| Dental Hospital of London, Leicester Sq., W.C. Free to the poor, together with any operative assistance that may be immediately necessary. For special operation, by Governor's letter | *Surgeons*, Messrs. Canton, Gregson, Hepburn, S. Bennett, Underwood, Woodhouse, Hern, Matheson, Parkinson, Read, Rogers, Truman. Daily, 9 to 11 |
| German Hospital. Free to Germans. See p. 16 | *Surgeons*, Messrs. A. P. Reboul, C. West, Th. 10 to 11 |
| Great Northern Central Hospital. Free. See p. 16 | *Surgeon*, Mr. E. Keen, W. 1.30 |
| Guy's Hospital. On payment of 3*d.* See p. 16 | *Surgeons*, Messrs. Moon, Pedley, Tu. Th. F. 12.30 |
| Hospital for Consumption, Brompton. By Governor's letter. | *Surgeon*, Mr. C. J. Noble, W., and when wanted |
| Hospital for Sick Children, Gt. Ormond Street, W.C. | *Surgeon*, Mr. Cartwright, W. 9 |
| King's College Hospital. See p. 16 | *Surgeon*, Mr. Cartwright, Tu. F. 10 |
| London Hospital. See p. 16 | *Surgeon*, Mr. A. W. Barrett, Tu. 9 |
| Metropolitan Free Hospital. Free. See p. 16 | *Surgeon*, Mr. G. Curnock, Tu. Th. S. 9 |
| Middlesex Hospital. See p. 16 | *Surgeons*, Messrs. Bennett, Hern, M. F. 9.30; W. 9 |
| National Dental Hospital, 149, Gt. Portland Street, W. By Governor's letter | *Surgeons*, Messrs. Williams, H. Rose, A. F. Canton, F. H. Weiss, T. Gaddes, A. Smith, W. G. Weiss, W. Humby, G. Bradshaw |
| National Hospital for Disease of Heart. By Governor's letter. | *Surgeon*, Mr. J. Fitzgerald, W. 11 |
| National Orthopædic Hospital, Gt. Portland St., W. | *Surgeon*, Mr. H. Harris |

